# Estimating the Per-Contact Probability of Infection by Highly Pathogenic Avian Influenza (H7N7) Virus during the 2003 Epidemic in The Netherlands

**DOI:** 10.1371/journal.pone.0040929

**Published:** 2012-07-13

**Authors:** Amos Ssematimba, Armin R. W. Elbers, Thomas J. Hagenaars, Mart C. M. de Jong

**Affiliations:** 1 Department of Epidemiology, Crisis Organization and Diagnostics, Central Veterinary Institute (CVI), Wageningen University and Research Centre, Lelystad, The Netherlands; 2 Quantitative Veterinary Epidemiology, Department of Animal Sciences, Wageningen University, Wageningen, The Netherlands; Duke-NUS Gradute Medical School, Singapore

## Abstract

Estimates of the per-contact probability of transmission between farms of Highly Pathogenic Avian Influenza virus of H7N7 subtype during the 2003 epidemic in the Netherlands are important for the design of better control and biosecurity strategies. We used standardized data collected during the epidemic and a model to extract data for untraced contacts based on the daily number of infectious farms within a given distance of a susceptible farm. With these data, we used a maximum likelihood estimation approach to estimate the transmission probabilities by the individual contact types, both traced and untraced. The estimated conditional probabilities, conditional on the contact originating from an infectious farm, of virus transmission were: 0.000057 per infectious farm within 1 km per day, 0.000413 per infectious farm between 1 and 3 km per day, 0.0000895 per infectious farm between 3 and 10 km per day, 0.0011 per crisis organisation contact, 0.0414 per feed delivery contact, 0.308 per egg transport contact, 0.133 per other-professional contact and, 0.246 per rendering contact. We validate these outcomes against literature data on virus genetic sequences for outbreak farms. These estimates can be used to inform further studies on the role that improved biosecurity between contacts and/or contact frequency reduction can play in eliminating between-farm spread of the virus during future epidemics. The findings also highlight the need to; 1) understand the routes underlying the infections without traced contacts and, 2) to review whether the contact-tracing protocol is exhaustive in relation to all the farm’s day-to-day activities and practices.

## Introduction

Highly Pathogenic Avian Influenza (HPAI) is one of the OIE listed poultry diseases. Several epidemics involving these viruses have occurred world-wide since its first description in northern Italy in 1878 [1], [2]. Examples of epidemics with devastating socio-economic consequences are the 1999 H7N1 epidemic in Italy [3] and the 2003 H7N7 epidemic in the Netherlands [4], [5]. Consequences of these epidemics include economic losses incurred in implementing control strategies and reduction in exports as well as a risk of spread to humans [6], [7]. The HPAI (H7N7) 2003 epidemic in the Netherlands involved 255 flocks; the virus was isolated in 241 of these flocks while the other 14 flocks were serologically positive [4], [5]. The majority of affected flocks were located in either of two areas with high poultry farm densities: one comparatively large area situated in the centre of the country, and one smaller area in the south; for more details we refer to Boender et al. [8].

Following the detection of the first outbreak, a control programme, as stipulated by the European Union, was implemented. This programme consisted of stamping out of infected flocks, movement restrictions and establishment of protection and surveillance zones. Despite additional control measures such as pre-emptive culling of flocks within a radius of 1 km of an outbreak and establishment of buffer zones between defined areas by complete depopulation of poultry flocks in these zones, there was a continued spread of the virus by mechanisms which are not clearly understood [5], [8], [9]. This spread only came to an end after the control measures had led to the culling of a large proportion of farms in the affected regions [5]. For the farmers, this meant incurring economic losses through and emotional burden of lost stock. Moreover, after a debate accruing from the 2001 Foot-and-Mouth Disease epidemic in the UK and the Netherlands, public opinion turned against the (large-scale) preventive killing of healthy animals; deeming it unethical [10], [11]. Hence the Dutch government is seeking alternative control measures to (large-scale) preventive culling, with emergency vaccination being the preferred strategy. However, in comparison with preventive culling, emergency vaccination would have the important disadvantage that its effect suffers from a 7 to 14 days protection delay [12]. This delay would prolong the time until epidemic control is obtained especially in the high density poultry areas (de Jong and Hagenaars [13] and the references therein). Thus the identification, testing and implementation of supplementary control strategies such as improved biosecurity are required. Identification of such strategies requires us to better understand the neighbourhood transmission (i.e., the indirect spread of the virus to farms neighbouring an infectious farm) of the virus.

Plausible mechanisms include movements of humans (professional and non-professional visitors, employees and farmers themselves), vehicular traffic (for example, delivery trucks), other fomites (such as tools, cell phones and shared farm equipment) and other vectors such as wind, rodents and insects [9], [14]–[17]. These transmission events involve transportation of the virus either in contaminated litter, faeces or skin and feathers that can colloid on the fomites or the vectors’ body. Therefore, in order to better control neighbourhood transmission, we need to understand deeper the steps involved in the whole virus dissemination process; a quite complex task.

Following potentially infectious contacts i.e. exposures, the probability of HPAI virus transmission may be contact-specific but will also depend on the contact patterns: i.e., the frequency of contacts and the contact network [18]–[20]. This interplay illustrates the need to determine the probability of virus transmission by a given type of contact during an epidemic. A combination of the estimated probabilities and the information on contact patterns can then be used to rank the individual contact risks and to assess risks of spread between different densely populated poultry areas. The resulting ranking is also important to guide further research and biosecurity implementation.

During the Dutch HPAI epidemic in 2003, the National Inspection Service for Livestock and Meat (RVV), responsible for the implementation of animal disease legislation and eradication of outbreaks of OIE listed diseases, was tasked with collecting epidemiological data and tracing of upward and downward contacts to and from infected farms. Using this data, Thomas and co-workers [9] performed a risk factor analysis to establish the factors that may have been responsible for the introduction of the virus on each of the farms involved. They found an increased risk of HPAI virus introduction in layer-finisher type poultry compared to other poultry types. Their analysis gave some clues on the risk factors for HPAI virus introduction such as poultry type and flock size. However, it is also important to gain insight into the transmission routes of the virus including the absolute risk of infection for given types of indirect contact between farms, an aspect addressed by the type of analysis we perform in this study. Since contact frequency and the per-contact probability of virus transmission partly determine the risk that a given category of contacts poses, the results of this analysis may facilitate a risk classification of these contacts. Such a classification is vital in the design of improved biosecurity and possibly other control strategies.

Our analysis aims to give quantitative insight into the role of the different between-farm contacts in the spread of the virus during an epidemic. We focus on the specific contacts that occurred during the HPAI (H7N7) epidemic in the Netherlands and estimate the probability of HPAI virus transmission attributable to each type of contact. Using published genetic data obtained by sequencing most of the samples collected during the epidemic [21], we assessed the consistency of our estimates with the genetic data. With these results, we provide scientific support to improve biosecurity measures to prevent transmission.

## Materials and Methods

### Data

We used two sets of data collected during the Dutch 2003 HPAI epidemic. One of the datasets was collected via a standardized field epidemiology investigation form of the RVV [22]. It included detailed information about day-to-day visits to all farms (infected and non-infected) such as visits for deliveries of farm inputs and for off-transport of outputs as well as professional and non-professional visits. In compiling this particular data, a follow-up to the visits mentioned by the farmers was made where possible. The preliminary data were cross-checked in detail and completed by the tracing unit of the crisis centre using the files obtained from the poultry-related businesses involved. This dataset captured information on a total of 614 visits originating from 203 infectious farms. Out of these visits, 381 were to infected farms. The total number of receiving farms was 325 of which 149 were ultimately infected. The other dataset was entirely about the visits that occurred in relation to measures aimed at controlling the epidemic (crisis organisation contacts). These included visits for: screening (i.e., the clinical inspection of poultry in the surveillance zone), tracing (i.e., the follow-up of visits from infected farms), indexing (i.e., the valuation of the flocks to be culled), and culling activities by the RVV [16]. From this dataset we selected visits to a farm that occurred up to seven days prior to and excluding its day of suspicion. For these contacts, we only considered same-day visits i.e., those that occurred on the same day that the person had visited an infectious farm.

In both datasets we could also find HPAI-related details such as the status and dates of clinical suspicion and stamping out for both the infected source farm and receiving farms. Since we could not identify a potentially infectious traced visit for all the ultimately infected farms, we introduced a category of ‘unknown’ contacts over different distance ranges. A farm was assigned one unknown contact per day when it was in the vicinity of an infectious farm. We chose three distance ranges (and hence three different unknown contact types) namely, 0–1 km, 1–3 km and 3–10 km of an infectious farm and assigned the unknown contacts accordingly. Details of these and all the other visits are given in Table 1.

**Table 1:** The description of the contacts extracted from the three datasets based on the assumed infectious and potential virus-introduction periods of this study.

Type of contact	Description
Feed delivery contact	A truck delivers feed to an infectious farm and proceeds to a susceptible farm.
Egg transport contact	A truck picks eggs or trays from an infectious farm and proceeds to a susceptible farm.
Rendering contact	A routine pick up of dead animals (not related to culling) occurred on an infectious farm and proceeds to a susceptible farm.
Other-professional contact*	A person (for example; veterinarian, dealer, advisor, technicians, and ‘unspecified-others’) visits an infectious farm and proceeds to a susceptible farm.
Crisis organisation contact	Person-contact for epidemic control activities such as screening, tracing, indexing, and culling that visited an infectious farm and proceeded to a susceptible farm.
Unknown contact:0–1 km**	Contact assigned to farm for every day that it is within 1 km of an infectious farm.
Unknown contact:1–3 km**	Contact assigned to farm for every day that it is between 1 and 3 km of an infectious farm.
Unknown contact:3–10 km**	Contact assigned to farm for every day that it is between 3 and 10 km of an infectious farm.

* The variable is a combination of related traced variables.

** A farm was assigned one unknown contact per day that it was in the vicinity of an infectious farm within the indicated distance range.

For each farm (infected or not) in the dataset, we extracted (and tabulated) all its exposures. In the summary table for the analysis, we indicated, for each contacted farm, the type and number of exposures as well as its ultimate status. A farm was deemed exposed if the visit occurred during the period when the virus was likely to have been introduced onto the receiving farm, here referred to as the potential virus-introduction period. Due to the uncertainty about the actual day of virus introduction, both the potential virus-introduction and infectious periods were assumed to begin seven days prior to the day of clinical suspicion, corresponding to the estimated farm infectious periods during the epidemic (i.e., 7.3 and 6.9 days for the two regions affected) for the period after epidemic detection [5]. The potential virus-introduction period lasted until the day before clinical suspicion while the infectious period lasted up to seven days after stamping out. This extended infectiousness was based on the hypothesis that the stamping out did not immediately rid the entire farm and its surroundings of all infectious material.

### Data Analysis

If 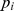 is the probability of infection per type 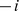 exposure, then the cumulative probability of a farm escaping infection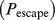 following a series of exposures is 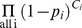 where 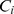 is the total number of type 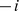 exposures and the compliment 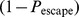 gives the probability of infection. In this case, we consider 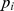 to be the conditional probability of virus transmission per contact i.e., the probability that a given contact transmitted the virus given that the contact occurred and that it originated from an infectious farm.

To estimate these probabilities, we used a maximum-likelihood approach. The likelihood function was given by
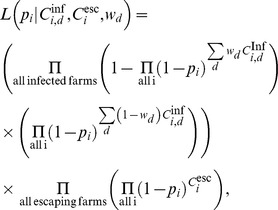
where 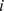 indexes the exposure-type, 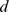 indexes the day-number (days before clinical suspicion day) that the contact occurred, 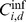 is the number of type 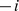 exposures to a case farm occurring 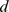 days before clinical suspicion, 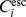 is the total number of type 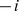 exposures to a non-case farm, 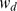 is the ‘weighting factor’ representing the probability that infection occurred through exposures occurring on day 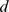 (see below), 
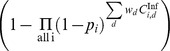
 is the probability of a farm being infected, 
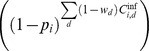
 is the probability of a farm escaping infection by type 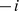 exposures, and 
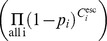
 is the probability of a farm escaping infection throughout the epidemic.

In this analysis, we assumed that: 1) the ‘exposure’ period started seven days prior to and lasted until the eve of clinical suspicion, 2) the infectious period began seven days prior to the day of clinical suspicion and lasted up to seven days after stamping out, 3) the conditional probability of infection was fully dependent on the contacts indicated in Table 1, and 4) the per-contact probability of infection by the traced contacts is independent of the distance between the source and receiving farms.

We used Mathematica 8 (Wolfram Research, Inc.) to perform the maximisation procedure. The 95% Confidence Intervals (CI) for the maximum likelihood estimates were computed using the likelihood ratio test. We quantified the contribution of the different contacts to the epidemic in terms of the number of new infections that they may have caused. This was obtained by multiplying their estimated per-contact probability with their frequency.

As an introduction can only occur on one day, we can only allow for the uncertainty about when this day was by giving weights to each of the possible introduction days with these weights adding up to one. For the base model, we used a uniform distribution to obtain 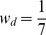. In other words, we assumed that each of the seven days of the probable period of virus introduction was equally likely to be the actual day of virus introduction. However, we also checked the outcomes based on different distributions in the sensitivity analysis.

### Sensitivity and Bias Analyses

#### Sensitivity analysis

We performed a sensitivity analysis to ascertain the effect, on the probability estimates, of the possible uncertainty in defining the distribution underlying the actual day of virus introduction over the assumed period. We performed this analysis by re-running the calculation with different distributions underlying the estimation of the weighting factor 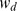. We assessed two other distributions in which the estimated weighting factors 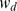 were adjusted to sum to one over the 7-day period, namely; 1) a distribution in which the probability is decreasing exponentially over the 7-day period at a rate determined by the survival of HPAI virus in manure (in this case 14 days [23]) and, 2) a unimodal distribution with the most likely day being 4 days prior to the day of clinical suspicion. In the second case, we used a discretized normal distribution with a truncated domain and 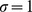 day. In both cases the assumed distributions were normalised to sum to one.

#### Potential difference in tracing efforts on case and non-case farms

We hypothesized that, during the epidemic, the tracing process may have been more rigorous on case farms compared to the non-case farms. We explored the effect of this possibility by considering a scenario where an under-representation of the contacts to the escaping farms – for example due to a more lax attitude of the tracing teams when on non-case farms – could have occurred. We estimated the maximum effect that this would have on the estimated probabilities in the following 3 steps: 1) if we let 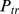 be the tracing probability of a contact, this would be the exact probability of tracing a contact to a non-case farm if no back-tracing at all was made at the non-case farm, 2) with back-tracing in place for the case farms, the probability of tracing their contact would be 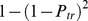 and finally, 3) the maximum bias due to under-representation occurs at the worst tracing level and would be given by 
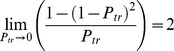
.

### Validation Against Genetic Data

In order to validate the estimated per-contact probabilities, we used the genetic data obtained by sequencing the majority of the samples collected for outbreak farms during the epidemic [21]. In this way, we used the genetic data to validate the estimated probabilities per contact: too few or too many genetic matches would cast doubt on the estimated probabilities. The approach developed for this validation is described below and in the Text S1.

With the contact inclusion criteria described under Data section, we extracted traced contact pairs, i.e. farm pairs (A, B) in which at least one contact originating from a then deemed infectious farm A to a hitherto susceptible (but ultimately infected) farm B, and occurring within the exposure period of farm B, was traced. We then used the genetic information generated from the majority of the samples taken from the affected farms during the epidemic as reported by Bataille et al. [21] in Figure S2 of their Supporting Information to identify which pairs had virus sequences for both farms. For those pairs (i.e., with complete genetic information), we compared their genetic sequences to ascertain which ones were sufficiently “matching” for transmission between A and B not to be unlikely. The number of genetically matching pairs, minus an estimate of the expected number of “by-chance” genetic matches, was then compared to the predicted number of pairs (amongst those with complete genetic information) in which virus transmission occurred (“transmission pairs”)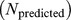. This number was estimated from the overall expected number by scaling it according to the expected contribution of the 28 contacts, relative to that of the 56, based on the estimated probabilities.

We considered four different (sets of) criteria for determining whether a contact farm pair (A, B) represents a genetic match. These (sets of) criteria differ in the level of genetic overlap required between the sequences from farm A and farm B to qualify as a genetic match. The most liberal criterion we considered was that all mutations in the virus of farm A compared to farm 1 (i.e., the first outbreak) were also found on farm B, i.e. when going from A to B no mutations are lost. This criterion is necessary because it is highly unlikely for the virus to lose mutations (i.e. undergo backward mutation) between source and receiving farms. In the other three, in addition to having no lost mutations, we permitted only a specific number/range of additional mutations: allowing no additional mutations at all, allowing ≤3 and, 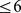 additional mutations. For each criterion, we calculated an expected number of transmission pairs by subtracting an estimate of the number of ‘chance matches’ from the total number of genetic matches (for details see Text S1).

## Results

With our selection criteria applied to the first dataset i.e., the data from the epidemiological investigation by the RVV, we were able to extract at least one traced exposure for 36 (i.e. 15%) ultimately infected farms and the number increased to 44 (i.e. 18%) upon including the crisis organisation contacts. With the complete dataset (i.e., the latter two together with the extracted unknown contacts), 227 (i.e. 94%) ultimately infected farms had been exposed. Thus with all the available and modelled data, all but 14 infected farms had either a traced exposure or it was in the neighbourhood of an infectious farm (unknown contacts).

In Table 1, we present a description of both the potentially infectious contacts recorded during the HPAI (H7N7) epidemic in the Netherlands in 2003 and the unknown contacts extracted for purposes of this study. In Table 2, we present the extracted number of contacts that met our inclusion criteria and their mean estimates of the per-contact probability of virus transmission (and their accompanying 95% CI). We also present in the same table the percentage (and 95% CI) of infections potentially caused by these contacts and the results of the sensitivity analysis.

**Table 2:** The number of contacts, the estimated per-contact transmission probabilities (95% CI), and the percentage of infections caused for the potentially infectious contacts during the HPAI (H7N7) epidemic in the Netherlands in 2003.

Contact type	Total number of contacts (to a case farm)	Per-contact probability of infection (95% CI)	Percentage of infections caused (% of 227 cases)	Sensitivity analysis: *w_d_*∼exponential decay function	Sensitivity analysis: *w_d_* ∼unimodal distribution
Unknown contact:0–1 km	27700 (3048)	0.0000570 (0.00–0.00044)	0.70 (0.00–5.37)	0.0000449	0.0000586
Unknown contact: 1–3 km	190846 (25035)	0.000413 (0.00031–0.00052)	34.72 (26.06–43.72)	0.000414	0.000430
Unknown contact: 3–10 km	1466564 (171021)	0.0000895 (0.000076–0.00010)	57.82 (49.10–64.61)	0.0000908	0.0000913
Crisis organisation contact	272 (16)	0.00110 (0.00–0.012)	0.13 (0.00–1.44)	0.000	0.000
Feed delivery contact	144 (23)	0.0414 (0.0043–0.085)	2.63 (0.27–5.39)	0.0342	0.0261
Egg transport contact	15 (8)	0.308 (0.16–0.48)	2.04 (1.06–3.17)	0.305	0.303
Other-professional contact	16 (5)	0.133 (0.023–0.29)	0.94 (0.16–2.04)	0.130	0.000
Rendering contact	12 (4)	0.246 (0.10–0.43)	1.30 (0.53–2.27)	0.239	0.179

Apart from the unknown and crisis organisation contacts, feed deliveries had the lowest per-contact probability of virus transmission of 0.0414 and potentially caused 2.63% of the new case farms while the egg transports had the highest per-contact probability of 0.308 and may have potentially caused 2.04% of the new case farms. The probability of virus transmission per crisis organisation contact was estimated to be 0.0011 and these visits may have caused 0.13% of the new case farms. The majority (92.54%) of the new cases were caused by the unknown contacts within the distance bands of 1–3 km and 3–10 km.

Analysing the sensitivity of the estimated probabilities to the assumed distribution underlying the actual day of virus introduction over the 7-day period, the outcomes from using the two alternative distributions (i.e., one with an exponentially decreasing probability and the other with unimodal distribution) were compared with those of the default distribution (i.e., uniform distribution). The estimates were very similar for most of the exposure types. The only differences found, but these were small, were in the per-contact probabilities for the crisis organisation contacts for both alternative distributions and the other-professional contacts for only the unimodal distribution (see Table 2). For both alternative distributions, the probabilities per crisis organisation contact were within the 95% CI of the default distribution whereas for the unimodal distribution, the per other-professional contact probability reduced from 13.3% to 0.0%. This reduction is a consequence of the very low weights *w_d_* assigned by the unimodal distribution to the days on which these contacts occurred. Three of the five contacts to ultimately infected farms occurred seven days prior to the day of clinical suspicion while the remaining two occurred four days and one day prior to the day of clinical suspicion.

With respect to the effect of the potential difference in tracing efforts on case and non-case farms – hence a possibility of under-representation of the contacts to non-case farms, we found that, with the worst tracing efforts, the contacts to case farms would be twice as likely to be traced as those to non-case farms. This implies that, at worst, the estimated probabilities could be double their ‘unbiased’ counterparts.

There were 56 traced contact pairs in which virus transmission may have occurred i.e. contacts from an infected farm to a newly infected farm. From the genetic data of the same outbreak [21], complete genetic information was available for 28 of these pairs (see Table S1). Using the estimated per-contact transmission probabilities and the numbers of each contact-type, we estimated that 15.96 outbreaks were explained by the traced contacts (Table 2). After rescaling, we obtained the predicted number of transmission pairs with matching genetic information 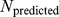 as 8.96. The lower and upper 95% confidence bounds of 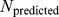 were estimated to be zero and 19 pairs respectively.

In Table S2, we present results of the pairwise genetic comparison of the 28 pairs for our different criteria of defining a genetic match. We observe (Table S2) that using the most strict criterion of requiring a ‘perfect’ genetic match between contact pairs (A, B) i.e., having no lost and no additional mutations when going from A to B, we estimated that virus transmission may have occurred in two pairs, reducing to 1.85 pairs upon subtracting the expected number of chance matches. If we defined a contact pair (A, B) to be a genetic match if there were no lost mutations when going from A to B and permitting any number of additional mutations, the number of transmission pairs was estimated to be nine, reducing to 7.23 pairs when adjusted for chance matching. Restricting the number of allowed additional mutations to 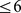 or to 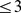 yields five matching pairs in both cases, reducing to 3.98 and 4.26 pairs respectively after subtracting the expected number of chance matches. All these results are within the 95% confidence bounds of the predicted number of transmission pairs with matching genetic information and hence the observed and predicted numbers are consistent.

## Discussion

The mechanisms of HPAI virus spread between farms are poorly understood; it has been hypothesized that the indirect between-farm contacts play a role [9], [14]–[17]. The frequency and the transmission effectiveness of these contacts determine their virus transmission rates. Here we perform a quantitative assessment of the contribution of indirect contacts to the spread of the virus between farms during the 2003 HPAI epidemic in the Netherlands. During this epidemic, potentially infectious contacts to both infected and escaping farms were traced. We use the collected data to quantify the per-contact probability of virus transmission between farms.

The estimated conditional probabilities of virus transmission are presented in Table 2. In terms of per-contact risk, the estimates reveal that egg transports have the highest risk with approximately 31% chance of transmission followed by the rendering visits with a chance of transmission of 25%. The unknown contacts in the distance band of 0–1 km have the lowest risk per contact although, as is clear from the 95% confidence bounds, its estimated per-contact probability is not significantly different from those of the other unknown contact categories. We expect that the implementation of preventive culling within 1 km of an infectious farm during the epidemic [5] has had a (strong) censoring effect on the detection of infected farms with 1 km of an infectious farm, thus producing a downward bias on the transmission probability per unknown contact within 1 km. We note that the estimated per-contact probability for the unknown contacts within the distance band of 1–3 km being higher than that of the 3–10 km distance band contacts reveals a distance-dependent transmission risk similar to the one found by Boender et al. [8].

Generally, most exposure-types (all except the crisis organisation contacts) made a substantial contribution to virus transmission during the epidemic. We note that the estimated per-contact probability of virus transmission by the crisis organisation contacts is 0.0011 and may have caused 0.13% of the infections. We note that when ignoring all other exposure types, i.e. considering the crisis organisation contacts alone in a separate analysis, we estimated a probability of 0.0327 per contact corresponding to 3.92% of the infections. This probability estimate is in agreement with the estimated maximum probability of virus transmission by a ‘control-person’ per visit of 0.037 reported by te Beest et al. [16] based also on a separate analysis of crisis organisation contacts only.

We hypothesize that the lower probability of infection per crisis organisation contact compared to that of the other-professional contacts which are almost of the same nature indicates that the epidemic control teams have better biosecurity than other visitors. The lower per-contact probability of infection per feed delivery compared to egg transport may be due to the difference in degree of contact and the re-use of egg trays. Unlike egg pick-up where the eggs have to be picked from the egg room, feed delivery may not involve accessing storage rooms or poultry houses. In most cases, the feed truck’s delivery tube is directly connected to the feed storage from the outside thereby reducing the risk of farm contamination.

In the sensitivity analysis, we find that the majority of the estimates are robust to the assumed distribution of the most likely day (among the seven days) of virus introduction. For the few sensitive (but less contributing) contact types, we concentrate on the results obtained using the uniform distribution as this assumes the least prior knowledge on the actual moment of disease introduction on the farm. Regarding the effect of a possible difference in tracing efforts on case and non-case farms, we have argued that an under-representation of the contacts to non-case farms may have at most doubled our probability estimates i.e., compared to the ‘ideal’ situation where the tracing efforts are the same for the case and non-case farms.

The pairwise comparison of the genetic information of the contact pairs (Tables S1 and S2) shows that the very low numbers of new infections explained by the traced contacts in our analysis is consistent with the genetic data. This genetic data has been used to construct transmission trees in reference [21] and in more detail in reference [24]. Our present analysis focused on estimating per contact transmission probabilities for the different between farm contact types using the contact tracing data. Note that there is no straight forward way to directly include genetic data in an estimation of the per contact transmission probabilities as the sequencing data only gives information on the case farms and not on the contact farms that escaped infection. However, both data types (i.e., genetic and epidemiological) can be combined within the same analysis to, for example, determine transmission pathways. This approach was proposed by Cottam et al. [25] in their analysis of part of the 2001 FMD epidemic in UK.

Stegeman and co-workers [18] performed a similar analysis on the 1997/1998 Classical Swine Fever (CSF) epidemic in the Netherlands. The common contact types in both studies are the ‘person’ (similar to ‘other-professional’) and rendering contacts. Perhaps remarkably, the estimated transmission probabilities for these contacts in our HPAI study are respectively two and four orders of magnitude higher than those estimated in the CSF study. These differences are mainly due to a difference in total numbers of between-farm contacts, with 16 and 12 for the HPAI epidemic (affecting 255 flocks) compared to 2468 and 10102 for the CSF epidemic (affecting 429 farms), respectively. The much higher numbers of contacts in the CSF epidemic are explained in part by the much longer duration of the epidemic: 15 months in comparison to the 3 months that the HPAI epidemic lasted. The difference in number of contacts is likely to be also related in part to the fact that the CSF epidemic was more spatially extended compared to the HPAI epidemic. As a result, there were more new outbreaks occurring outside existing stand-still areas (in which onward contacts are more restricted) for the CSF epidemic as compared to the HPAI epidemic.

With our contact inclusion criteria, 44 infected farms have at least one traced exposure i.e., excluding the ‘unknown’ contacts. The outbreaks that could not be linked to any known potentially infectious contact may not only be attributed to the inability to trace all targeted contacts. Rather, they may serve as a hint about the presence of other (un-targeted and hence untraced or even untraceable) mechanisms. This highlights the need to better understand the possible mechanisms of untraced transmission.

It is important to realize that the probabilities estimated are conditional on the contact originating from an infectious farm and do not represent the actual risk of HPAI virus transmission by these contacts during the epidemic. We also emphasize that care should be taken when interpreting the per-contact probability estimate for the rendering contacts due to the possible correlation between this contact-type and the increased mortality which could have occurred during the silent spread period of the virus on the farm i.e., the virus could have already been circulating undetected on the receiving farms. Nevertheless, the probability estimates together with the risk-based ranking for the different contacts obtained in this study can help design better control strategies against HPAI virus transmission between-farms by these contacts.

All in all, after estimating the per-contact probability of virus transmission for the different contacts, we conclude that all the identified contacts made a substantial contribution to the risk of virus transmission between farms. Therefore, any measures to reduce on their frequency and to improve biosecurity during all these contacts are potentially worthwhile. The fact that the ‘unknown’ contacts contributed the most (causing 93.24% of the infections among themselves) emphasizes the need for a better understanding of the mechanisms underlying virus transmission.

The findings of this study contribute to the greatly desired understanding of the mechanisms of indirect transmission of HPAI virus between farms. Our results suggest that, apart from the unknown contacts, egg delivery contacts are interesting targets for improvements in biosecurity due to their high per-contact probability (31%) in infecting the receiving farms. They further suggest that the biosecurity applied to the crisis organisation contacts seems to be adequate at least for preventing the persons themselves from becoming important fomites between registered visits. Overall, these findings provide a scientific basis to conduct further studies, epidemiological or otherwise, to evaluate the impact of improved biosecurity and minimized contact-frequency in controlling the between-farm spread of HPAI virus during epidemics. The knowledge gained in this study can further be supplemented by research aimed at disentangling the ambiguous category of ‘unknown’ contacts defined in this study.

## Supporting Information

Table S1 **Summary of genetic differences between isolates from the source and receiving farms for the traced contact pairs.**
(DOC)

Table S2 **The observed number of genetically matching pairs (A, B), within 28 pairs of outbreak farms linked by traced contacts, for different criteria of defining a genetic match.**
(DOC)

Text S1 **Further details on the validation against genetic data.**
(DOC)
