# Supplementary material for: Estimating the Per-Contact Probability of Infection by Highly Pathogenic Avian Influenza (H7N7) Virus during the 2003 Epidemic in The Netherlands
Source: PLoS One. 2012 Jul 13;7(7):e40929. doi: 10.1371/journal.pone.0040929 (PMC3396644; doi:10.1371/journal.pone.0040929)
Supplement: Table S1 — Summary of genetic differences between isolates from the source and receiving farms for the traced contact pairs. (DOC) [file pone.0040929.s001.doc]

**Table S1.** Summary of genetic differences between isolates from the source and receiving farms for the traced contact pairs

| Contact pairs (source, receiver) | Exposure type | Additional mutations^$^ | Lost mutations ^$^ |
| --- | --- | --- | --- |
| (001,007) | Rendering visit |  | ** |
| (001,010) | Rendering visit | 3 | 0 |
| (001,022) | Rendering visit | 3 | 0 |
| (013,079) | Rendering visit |  | * |
| (003,098) | Egg transport |  | *** |
| (010,080) | Egg transport | 4 | 3 |
| (018,098) | Egg transport |  | ** |
| (046,052) | Egg transport | 2 | 1 |
| (046,061) | Egg transport | 0 | 0 |
| (027,036) | Egg transport | 7 | 1 |
| (034,033) | Egg transport |  | * |
| (027,082) | Egg transport | 8 | 1 |
| (022,034) | Feed delivery |  | ** |
| (091,129) | Feed delivery | 9 | 0 |
| (117,081) | Feed delivery |  | ** |
| (076,114) | Feed delivery |  | ** |
| (090,161) | Feed delivery | 1 | 2 |
| (091,140) | Feed delivery | 11 | 0 |
| (106,127) | Feed delivery |  | *** |
| (113,166) | Feed delivery | 1 | 0 |
| (030,061) | Feed delivery | 2 | 5 |
| (091,145) | Feed delivery | 10 | 0 |
| (113,180) | Feed delivery | 12 | 5 |
| (013,125) | Feed delivery |  | * |
| (016,053) | Feed delivery |  | ** |
| (214,234) | Feed delivery |  | Deletion |
| (093,177) | Feed delivery |  | * |
| (221,232) | Feed delivery |  | Deletion |
| (090,122) | Feed delivery |  | ** |
| (043,106) | Feed delivery |  | ** |
| (124,156) | Feed delivery |  | * |
| (094,090) | Feed delivery | 8 | 4 |
| (124,189) | Feed delivery |  | *** |
| (106,149) | Feed delivery |  | *** |
| (106,149) | Feed delivery |  | *** |
| (003,030) | Other-professional visit |  | * |
| (016,030) | Other-professional visit | 5 | 2 |
| (005,043) | Other-professional visit | 0 | 0 |
| (155,154) | Other-professional visit | 2 | 1 |
| (133,200) | Other-professional visit |  | *** |
| (051,073) | Crisis organisation contact |  | ** |
| (108,073) | Crisis organisation contact |  | ** |
| (023,033) | Crisis organisation contact | 8 | 1 |
| (001,033) | Crisis organisation contact | 10 | 0 |
| (131, 171) | Crisis organisation contact | 6 | 11 |
| (128, 171) | Crisis organisation contact |  | * |
| (097, 083) | Crisis organisation contact |  | *** |
| (117, 097) | Crisis organisation contact |  | ** |
| (117, 097) | Crisis organisation contact |  | ** |
| (051, 108) | Crisis organisation contact | 5 | 3 |
| (053,108) | Crisis organisation contact |  | * |
| (054,108) | Crisis organisation contact | 10 | 5 |
| (149,169) | Crisis organisation contact |  | * |
| (107,110) | Crisis organisation contact | 4 | 3 |
| (105,123) | Crisis organisation contact | 11 | 6 |
| (102,123) | Crisis organisation contact | 3 | 2 |

* sample from source farm was not yet sequenced

** sample from receiving farm was not yet sequenced

*** samples from both receiving and source farms were not yet sequenced

^$^ Comparison is between the virus on recipient farm and that on the sender farm using virus on farm 001 as the root, i.e. additional mutations are the number of mutations that the sender farm’s virus is closer to the root than the recipient farm’s virus. In addition the recipient farm’s virus may differ from the sender farm’s virus because of mutations that the sender farm’s virus had and the recipient farm’s virus has lost. If the number of mutations lost is equal to zero, the sender farm and the recipient farm are on the same branch with the sender being closer to the root. We assume that when the virus on the sender farm has a deletion the virus on the recipient farm cannot be without that deletion so this combination cannot be a transmission event. The mutations are counted based on Figure S2 of Bataille et al. (2011).
