# Supplementary material for: Estimating the Per-Contact Probability of Infection by Highly Pathogenic Avian Influenza (H7N7) Virus during the 2003 Epidemic in The Netherlands
Source: PLoS One. 2012 Jul 13;7(7):e40929. doi: 10.1371/journal.pone.0040929 (PMC3396644; doi:10.1371/journal.pone.0040929)
Supplement: Table S2 — The observed number of genetically matching pairs (A, B), within 28 pairs of outbreak farms linked by traced contacts, for different criteria of defining a genetic match. (DOC) [file pone.0040929.s002.doc]

**Table S2.** The observed number of genetically matching pairs (A, B), within 28 pairs of outbreak farms linked by traced contacts, for different criteria of defining a genetic match. Our analysis of transmission probabilities of contact types predicts transmission to occur from A to B for an expected number $N_{\text{predicted}}$of 8.961 pairs within the 28 (due to the traced contacts). This number is obtained by multiplying the expected number of transmission pairs (which is 15.96) by a scaling factor of 0.5615. This scaling factor is the expected contribution of 28 contacts relative to that of the 56 based on a weighted count of the contacts of each type (given in Table S1), using the per-contact transmission probabilities as weights.

|  | No lost mutations in B compared to A | No lost mutations AND ≤6 additional mutations in B compared to A | No lost mutations AND ≤3 addition mutations in B compared to A | No lost mutations AND no additional mutations in B compared to A |
| --- | --- | --- | --- | --- |
| #matching pairs (out of the 28 pairs); M | 9 | 5 | 5 | 2 |
| Counts of pairs meeting criterion; n_c | 2125 | 1231 | 886 | 182 |
| ‘probability of chance agreement’; p_c=n_c/(184x183)^$^ | 0.06311 | 0.0366 | 0.0263 | 0.00541 |
| Expected number of chance matches; R=p_c*28 | 1.77 | 1.02 | 0.74 | 0.15 |
| Matching pairs corrected for chance (M-R) | 7.23 | 3.98 | 4.26 | 1.85 |

^$^ The total number of pairs possible is 184×183 as there are 184 outbreak farms for which sequencing information is available and for any two farms A and B, the pair (A, B) is different from (B, A).
