# Supplementary material for: Estimating the Per-Contact Probability of Infection by Highly Pathogenic Avian Influenza (H7N7) Virus during the 2003 Epidemic in The Netherlands
Source: PLoS One. 2012 Jul 13;7(7):e40929. doi: 10.1371/journal.pone.0040929 (PMC3396644; doi:10.1371/journal.pone.0040929)
Supplement: Text S1 — Further details on the validation against genetic data. (DOC) [file pone.0040929.s003.doc]

**Estimating the per-contact probability of infection by highly pathogenic avian influenza (H7N7) virus during the 2003 epidemic in the Netherlands**

**Supporting Information file Text S1**

Amos Ssematimba^1,2^, Armin R.W. Elbers^1^, Thomas J. Hagenaars^1^, Mart C.M. de Jong^2^

^1^Department of Epidemiology, Crisis organization and Diagnostics, Central Veterinary Institute (CVI) part of Wageningen University and Research Centre, Lelystad, The Netherlands

^2^Quantitative Veterinary Epidemiology, Department of Animal Sciences, Wageningen University, Wageningen, The Netherlands

**Further details on the validation against genetic data**

***Number of chance matches***

The number of chance matches was estimated as follows: from Figure S2 of Bataille et al., we counted the total number of possible outbreak farm pairs that met a specific criterion for defining a genetic match. We then divided the outcome by the total number of possible outbreak farm pairs to obtain the probability of having a matching pair just by chance. By multiplying this probability with the number of contact pairs with complete genetic information, we obtained the expected number of chance-matches.

***Confidence bounds for the predicted number of genetic matches***

The 95% confidence bounds of $N_{\text{predicted}}$ were calculated based on a ‘mean’ per-contact probability $\left(P_{\text{combined}}\right)$ and its lower $\left(P_{\text{combined}}^{L}\right)$ and upper $\left(P_{\text{combined}}^{U}\right)$ 95% confidence bounds by grouping all the traced contacts into one category and re-running the analysis described under Data analysis section in main text. Then the estimated $P_{\text{combined}}^{L}$ and $P_{\text{combined}}^{U}$, after multiplication by the probability of having a pair with complete genetic information, were each used as probabilities of a binomial distribution for the number of observed genetic matches, with the total number of traced contact as the binomial total. The 2.5 percentile of the binomial distribution corresponding to $P_{\text{combined}}^{L}$ and 97.5 percentile of that corresponding to $P_{\text{combined}}^{U}$ gave the 95% confidence bounds of $N_{\text{predicted}}$.

**References**

1. Bataille A, van der Meer F, Stegeman A, Koch G (2011) Evolutionary Analysis of Inter-Farm Transmission Dynamics in a Highly Pathogenic Avian Influenza Epidemic. PLoS Pathog 7: e1002094.
